# Numerical optimization of alignment reproducibility for customizable surgical guides

**DOI:** 10.1007/s11548-015-1171-8

**Published:** 2015-04-11

**Authors:** Thomas Kroes, Edward Valstar, Elmar Eisemann

**Affiliations:** 1 Computer Graphics and Visualization Group, Department of Intelligent Systems, Delft University of Technology, Mekelweg 4, 2628 CD Delft, The Netherlands; 2 Department of BioMechanical Engineering, Delft University of Technology, Mekelweg 2, 2628 CD Delft, The Netherlands; 3 Biomechanics and Imaging Group, Department of Orthopaedics, Leiden University Medical Center, Albinusdreef 2, 2333 ZA Leiden, The Netherlands

**Keywords:** Knee replacement surgery, Physical simulation, Customizable surgical guide, Surgical navigation device, Genetic optimization

## Abstract

**Purpose:**

Computer-assisted orthopedic surgery aims at minimizing invasiveness, postoperative pain, and morbidity with computer-assisted preoperative planning and intra-operative guidance techniques, of which camera-based navigation and patient-specific templates (PST) are the most common. PSTs are one-time templates that guide the surgeon initially in cutting slits or drilling holes. This method can be extended to reusable and customizable surgical guides (CSG), which can be adapted to the patients’ bone. Determining the right set of CSG input parameters by hand is a challenging task, given the vast amount of input parameter combinations and the complex physical interaction between the PST/CSG and the bone.

**Methods:**

This paper introduces a novel algorithm to solve the problem of choosing the right set of input parameters. Our approach predicts how well a CSG instance is able to reproduce the planned alignment based on a physical simulation and uses a genetic optimization algorithm to determine optimal configurations. We validate our technique with a prototype of a pin-based CSG and nine rapid prototyped distal femora.

**Results:**

The proposed optimization technique has been compared to manual optimization by experts, as well as participants with domain experience. Using the optimization technique, the alignment errors remained within practical boundaries of 1.2 mm translation and $0.9^\circ$ rotation error. In all cases, the proposed method outperformed manual optimization.

**Conclusions:**

Manually optimizing CSG parameters turns out to be a counterintuitive task. Even after training, subjects with and without anatomical background fail in choosing appropriate CSG configurations. Our optimization algorithm ensures that the CSG is configured correctly, and we could demonstrate that the intended alignment of the CSG is accurately reproduced on all tested bone geometries.

## Introduction

Osteoarthritis and rheumatoid arthritis lead to irreversible damage to joints. These conditions impact the patients’ mobility and lead to severe pain. An orthopedic surgeon can replace the joint in order to reduce these symptoms. During joint replacement surgery, the shape of the bone has to be altered (by sawing and drilling) in such a way that the orthopedic implant can be securely installed into the planned position and orientation. There are many factors, such as blood loss, aseptic loosening, and operating time, which can have a negative impact on the patient’s treatment. Among these factors is mal-alignment, which has an important effect on the stability of the implant and in some cases also the functioning of the joint, e.g., range of motion [3, 11]. In this work, we will focus on this particular aspect. With the conventional array of surgical instruments, implant alignment is a challenging task, because anatomical references, used for implant alignment, are obscured by layers of tissue, such as muscles and fat.

Alignment accuracy can be improved using CAOS systems that provide planning routines and active/passive guidance during joint replacement procedures [8, 10, 14, 28, 30].

However, most CAOS systems tend to increase operating time and add complexity to the surgical procedure. They have a steep learning curve, and the accuracy depends on the quality of the input information, e.g., reconstructed bone, quality of marker tracking, and registration [23]. At the same time, this type of navigation requires auxiliary hardware, which needs to be sterilized. Furthermore, a recent meta-study shows that the increased accuracy of implant alignment does not lead to improved postoperative function recovery [35].

On the contrary, PSTs are surgical guides that fit uniquely on a patient and are manufactured using rapid prototyping technology. They encode the complete planning in the template and provide guidance during pedicle screw insertion [4, 17, 27, 32], knee [5, 20, 21], hip [1, 19] and shoulder replacement [15]. No specialized auxiliary hardware is needed for navigation, but a surgeon can also not make adjustments to the planning during the procedure. This aspect can be problematic should the template not fit correctly due to manufacturing issues, poor handling of the template, and/or poor 3D reconstruction of the bone, on which the template planning is based. These templates can only be used once, after which they are disposed of.

CSGs attempt to mitigate the problems associated with existing CAOS approaches, such as mal-alignment [9]. The CSG is a mechanically adjustable surgical instrument that fits uniquely onto a patient and is designed to provide guidance (e.g., holes for drilling and slits for cutting) to the surgeon during surgery. In contrast to PSTs, this type of device is reusable, but needs to rely on a manual configuration step. While this feature even makes it possible to apply changes to the surgical plan during an actual procedure, it is often a complex task and very difficult to perform correctly by hand.

**Fig. 1:**
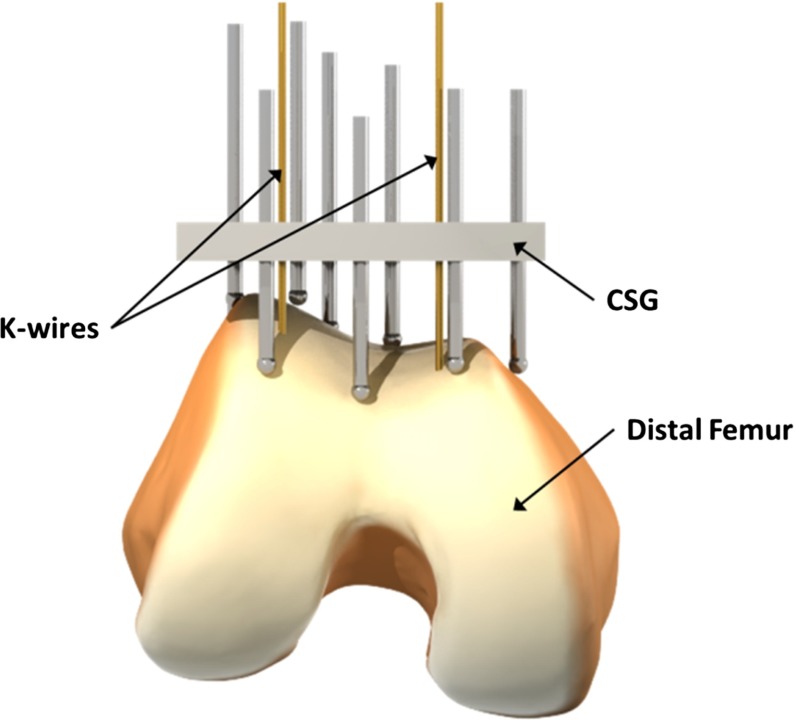
The pin-based version of the CSG applied to the distal femur. The surgical plan is transferred to the operating theater by encapsulating the shape of the bone in the guide using a collection of strategically distributed pins (which collide with the surface of the bone). The CSG has predefined holes for the k-wires that are compatible with standard instrumentation for performing the principal bone cut

The objective of this article is to investigate how to automate the CSG configuration process for an arbitrary CSG. We illustrate our method by applying it to knee replacement surgery. Via a semiautomated planning step, the CSG becomes patient specific and ensures that the planned alignment can be accurately reproduced and the device snaps into the intended position and orientation (see Fig. 1). To this extent, we created a novel and generic computer-assisted planning method that predicts the CSG trajectory to the bone and its stability and guides the configuration process. The method is designed to allow users to indicate particular regions on the bone to be avoided (for instance due to bone spurs). We validate our optimization method via a simulation, as well as a real-world setting with a pin-based CSG applied to a rapid prototyped bone model.

The remainder of this paper is structured as follows: After the discussion of related work, we briefly describe the involved material and the exemplary CSG design used throughout this article. We then present our algorithmic solution to configure the device for a specific patient. Finally, we present the results of our approach using rapid prototyping and illustrate its usefulness in the context of joint replacement surgery, before concluding.

## Related work

In the field of CAOS, only few CSG-oriented publications exist, which we mention here. A CSG for hip replacement surgery has been introduced in [29] in order to improve acetabular cup positioning. A novel method to transfer a computer-assisted knee replacement surgery to the patient, using an adjustable pin-grid-based jig, is described in [6]. The results of a pilot study conducted on the distal femur show a relatively high axial translation error, which might relate to the fact that the pin configurations were manually configured, an issue we address in this work.

In [33], a drill guide for dental implants is described. In this approach, a set of actuated pins is used to register the instrument to the bone and to reproduce the planned implant direction. In contrast to our method, pins with sensors are used to obtain a shape-based registration [24–26], whereas in our solution the pin position and layout are fixed and the insertion depth determined a priori. In [36], a robot-assisted drill guide is described that uses a special registration process that allows surgeons to drill holes along a predefined axis. Additionally, an analytical method for calculating the docking robustness of PSTs in 2D has been developed [13]. Another method analyzes patient CT scans and identifies bone surface regions where the contact adds the most to stability [22]. This input could be integrated in our approach.

## Materials and methods

### Pin-based CSG

**Fig. 2:**
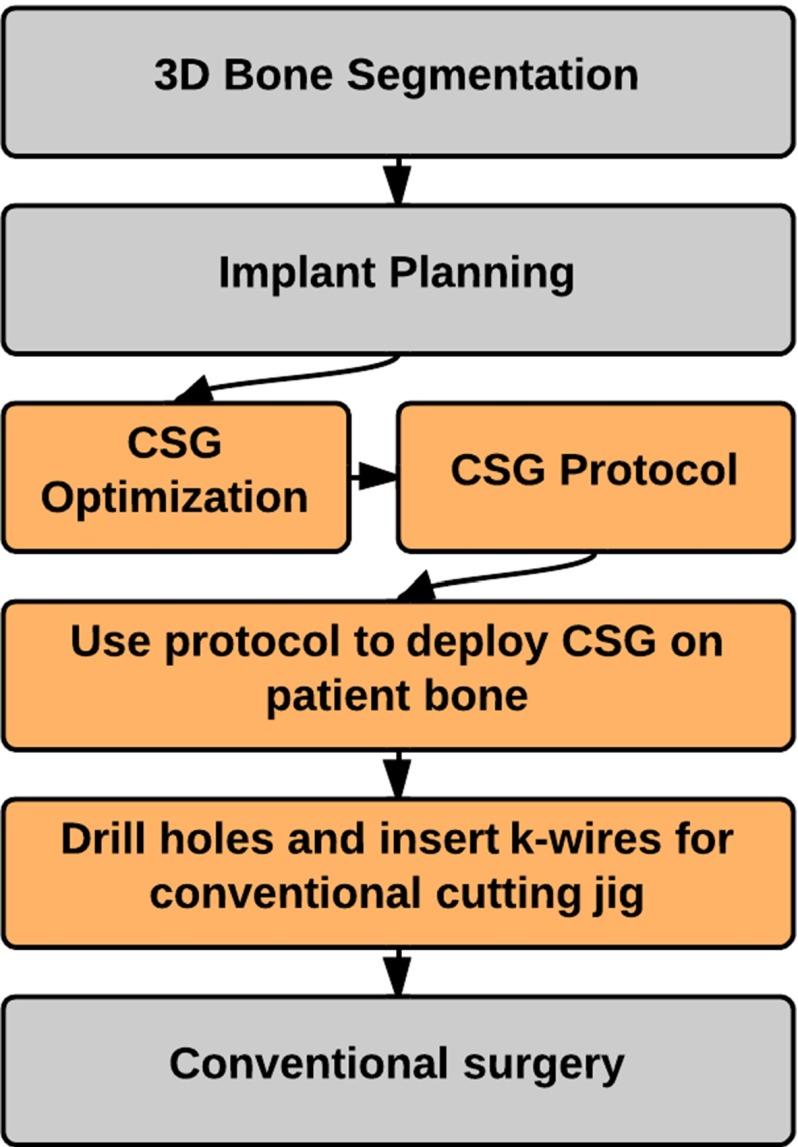
CSG pipeline for knee replacement surgery. The $orange\,steps$ are specific to the CSG. The CSG takes as input the planned implant alignment and uses it to make the CSG patient specific and to optimize its configuration. When the CSG is optimized, its configuration protocol is used intra-operatively to adjust the CSG and to dock it on the patients’ bone. Next, holes are drilled for k-wires. Once the k-wires are inserted, a cutting block can be attached to the k-wires and conventional surgery can take over

The pin-based CSG studied in this paper is inspired by [6] and uses a sparse point-surface contact set (a selected number of strategically placed adjustable pins) to achieve a stable configuration between the device and the bone. The pin-based CSG consists of a regular grid of holes, through which pins can be inserted (see Fig. 1). To give a more precise impression of how our method should be integrated in the surgical pipeline, we give an overview for a CSG-assisted total knee replacement in Fig. 2.

Since the CSG works on the basis of sparse point-surface contact, it is of paramount importance to configure the device appropriately. First, the pin depth should be adjusted in such a way that all pins touch the bone surface when the device is in its intended position. Further, their number should stay reasonable for a clinical setting, which implies that they need to be strategically distributed. Given these constraints, our algorithm derives an optimal set of active pins csg, a CSG configuration, via a simulation and optimization procedure (see Fig. 1).

**Fig. 3:**
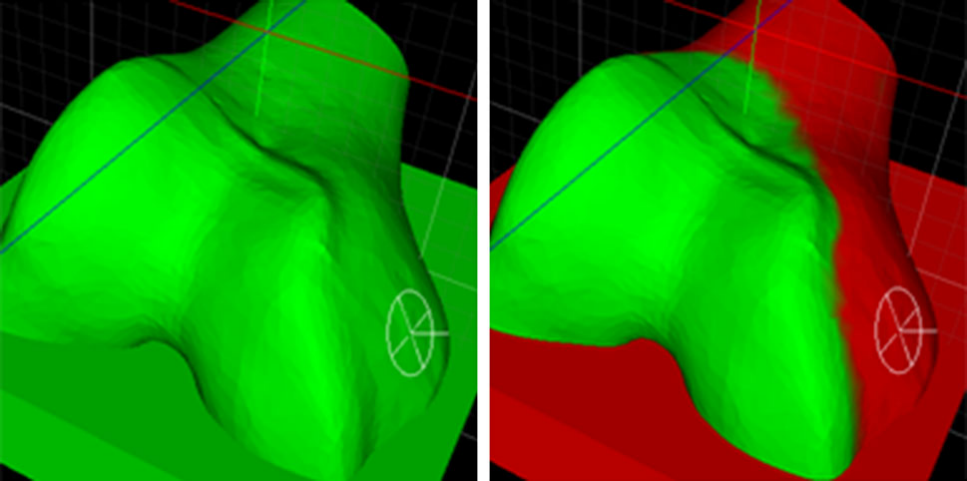
$\textit{Left}$ full exposure, pins can be deployed anywhere on the bone/cartilage. $Right$ limited exposure. The orthopedic surgeon paints the areas on the bone that are deemed accessible during surgery, thus limiting where pins can be deployed

In our pilot study, we fabricated a prototype of the pin-based CSG, which consists of a square plate (width $=$ 90 mm, height $=$ 10 mm, depth $=$ 90 mm) with $11\,\times \,11$ holes (radius $=$ 2 mm), through which pins (length $=$ 100 mm, $\mathrm{radius_\mathrm{tip}}=$ 2.5 mm) can be inserted and fixated, see Fig. 1. The prototype of the pin-based CSG merely serves as a tool to validate our optimization method and is not directly intended for clinical use. The pin-based CSG can contain 121 pins in total; however, it seems impractical to adjust all pins. Setting a single pin takes at least 10 s, and the manual configuration process is increasingly tedious and cumbersome with a larger number. Additionally, sometimes it is important to avoid placing pins, which would lead to unwanted contacts with certain regions of the bone, e.g., those designated inaccessible by the surgeon (see Fig. 3). We will refer to these situations as full, respectively, limited exposure.

Given the limit on the number of pins, the amount of possible distributions, and the complexity of the physical interactions between the CSG and the bone, it is challenging to configure the CSG to ensure a very low rotational and translational error after application to the bone. Using our algorithm, this configuration step can be automated, leading to a small number of strategically positioned pins, ensuring stability and accuracy.

### CSG optimization

The core of our optimization method is the derivation of the CSG configuration, which we will describe in detail in this section. First, we define the CSG objective function to measure the device’s deviation from its intended location, while considering an uncertainty in the CSG placement process. We then explain how this objective function is minimized with the help of a genetic algorithm in order to optimize the configuration of the CSG. For convenience, Table 1 contains an overview of all the variables used in this section.

**Table 1:** Optimization variables

$s$	Bone surface
$M_\mathrm{d}$	Maximum deviation over all pins in a CSG
$T$	Pin number optimization threshold applied to $M_\mathrm{d}$
$n_\mathrm{csg}$	CSG number of pins
$n_\mathrm{max}$	CSG maximum number of pins
$d$	Docking movement (origin and direction)
$E_\mathrm{csg}$	CSG error
$E_\mathrm{d,\mathrm csg}$	CSG alignment error after docking
$n$	Population size
$c_\mathrm{e}$	Elite CSG percentage
$c_\mathrm{c}$	Crossover probability
$c_\mathrm{m}$	Mutation probability
$c_\mathrm{n}$	New random CSG probability
$i$	Stop if not improving after $i$ iterations

#### CSG objective function

In order to steer the optimization method toward a suitable CSG configuration, an appropriate CSG objective function is key. Ultimately, it should be an indicator of how well the device snaps into its intended position and how stable it is. Hence, the baseline of our objective function is a measurement of the alignment error (global drift and orientation deviation) when the device reached an equilibrium state on the bone. Nonetheless, as angles and translational movement are not comparable, we opt for an objective function which allows us to bound both.

**Fig. 4:**
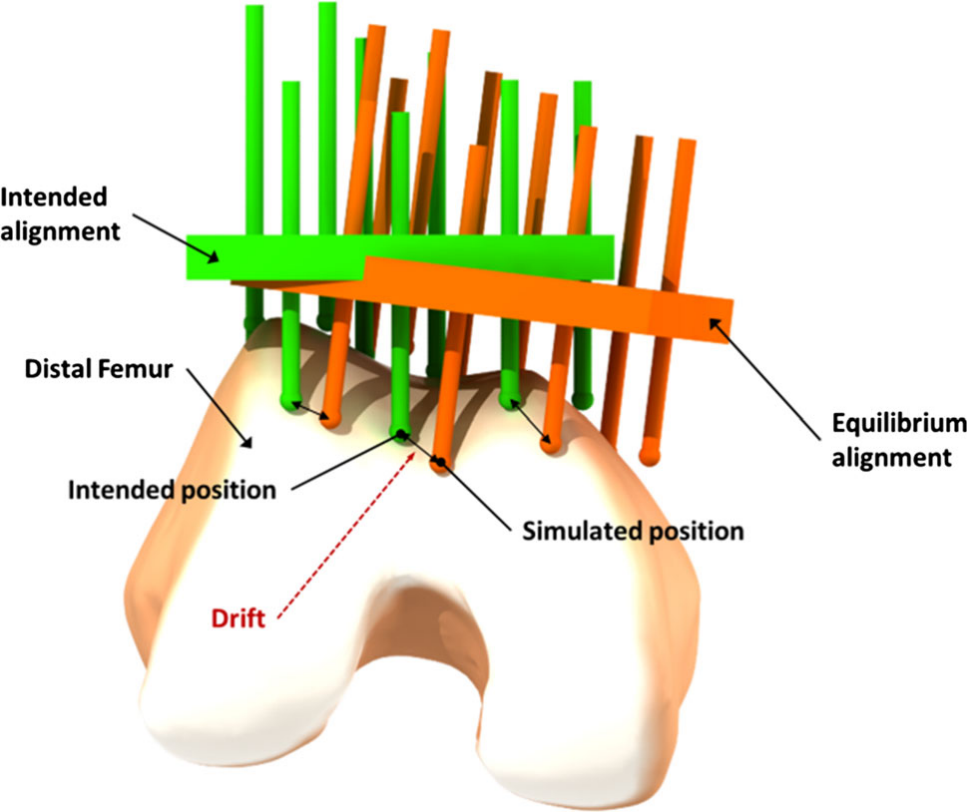
The drift value for a single pin is defined as the Euclidean distance between the intended pin position and the simulated pin position at equilibrium

Assuming for the moment only a single direction-origin pair $d$ defining a translational movement toward the bone surface, we then define for a given CSG configuration (csg) the CSG error for $E_\mathrm{d,\mathrm csg}$ as the maximum deviation $M_\mathrm{d}$ over all pins. In other words, we compute $M_\mathrm{d}$, as the maximum Euclidean distance between the intended and actual pin location (see Fig. 4). Given $M_\mathrm{d}$, we can derive a bound on global drift and orientation deviation and vice versa. In our work, we impose a maximally acceptable drift of 1.5 mm, which implies a rotational error of $<$1$^{\circ }$ (see Figs. 15, 16). The surgeon can also modify this value prior to surgery.

**Fig. 5:**
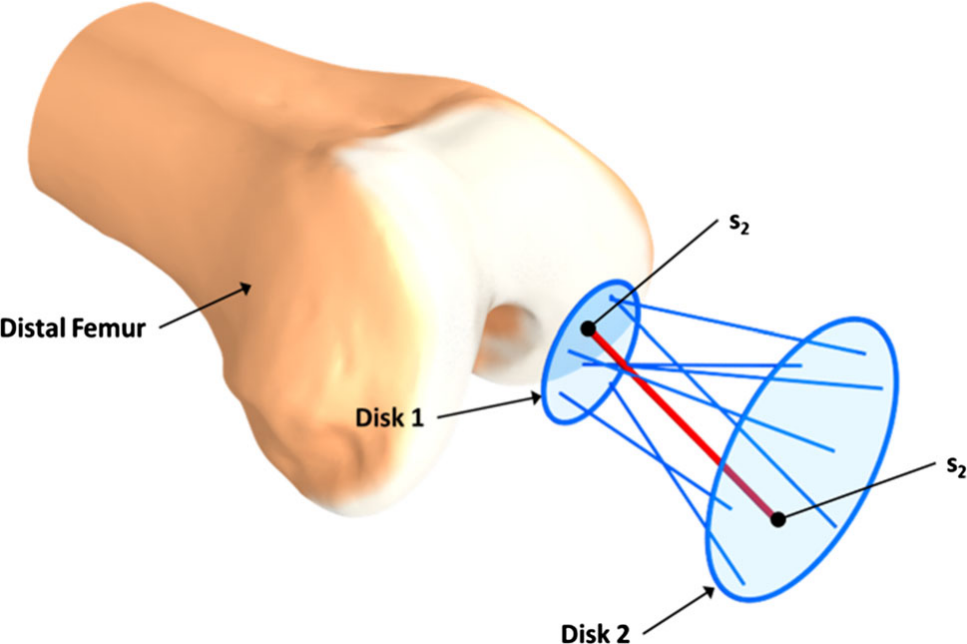
Docking directions are generated inside a truncated cone by picking a random point on Disk 1 and 2, these two points ($s1$ and $s2$) are then connected and form the docking direction $d$. The default radius for Disk 2 is 5 mm, and the cone angle is $15^{\circ }$. The $cone\, angle$ represents the placement uncertainty, and does not dependent on the size of the patient. However, this parameter can be changed by the user prior to optimization

One important observation is that the equilibrium state of the CSG depends on the bone surface $s$ and a surgeon would not be able to move a device perfectly along a single direction. Consequently, several docking movements $d$, in the form of a starting position and direction, should be tested. In practice, we restrict $d$ to a truncated cone (see Fig. 5). The directions and origins inside the truncated cone are tested; the final objective function is then $E_\mathrm{csg}=\mathrm{max}_\mathrm{d \in cone}E_\mathrm{d,\mathrm csg}$. In practice, we use 40 directions because the maximum drift parameter changed only marginally (drift $<$0.05 mm) hereafter and the computational overhead of adding more directions does not pay off in this case.

**Fig. 6:**
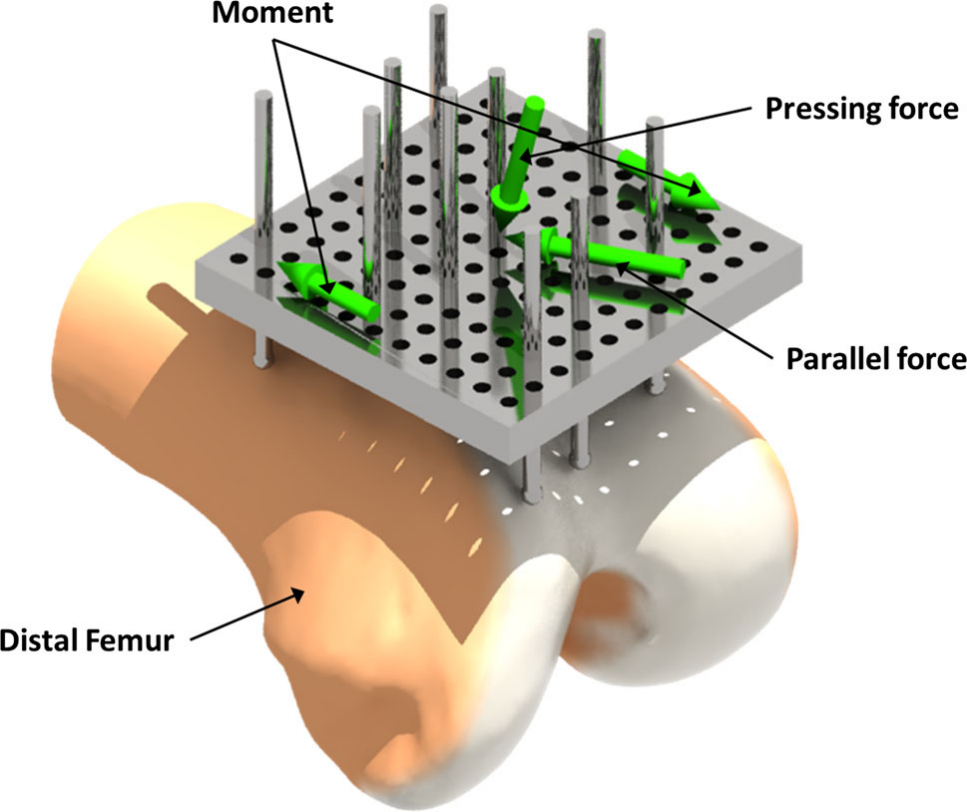
Visualization of the CSG and the external forces applied during the physical simulation. The moment magnitude varies periodically with a sine function, the parallel force rotates around the center of the CSG

To determine the CSG equilibrium state, we employ a physical simulation that predicts how the device will behave. During the simulation, we subject the CSG to external forces to mimic the real behavior of the docking process. We observed that apart from the principal pressing force along $d$, the user will exert moments and parallel forces on the CSG in an attempt to assess its stability using the haptic feedback that it provides (if the CSG wanders under these external forces, it is not securely docked in the right position). Taking the pressing force into account is useful because the morphology of the host bone might make particular pressing directions more suitable. For instance, in the case of the distal femur, we observed that when applying the pressing force under a slight angle, the CSG behavior improves (see Fig. 6). In most cases, the CSG will reach an equilibrium state in which the CSG error can be determined. However, in some cases, the CSG will simply fall off and the physical simulation will be aborted prematurely. Here, we consider the error to be infinite, indicating that it is not useful.

The objective function also allows us to take several constraints into account. First, the truncated cone defining possible values for $d$ can be manually modified by the surgeon to adjust the angle of approach and the cone angle. For instance, a right-handed surgeon might never place the device from the left, due to limited exposure or the way that the patella is exposed. Such adjustments can be performed via a simple interface showing the virtual bone and the cone. The cone angle range is limited to $10^{\circ }$–$30^{\circ }$ in order to prevent unreasonable docking directions (e.g., from below the surface). In practice, these constraints are easily fulfilled. Additionally, we provide standard settings to add an automatic bias of a $5^{\circ }$ inclination for left/right-handedness of the surgeons, but refrained from using it in our study to avoid such prior knowledge.

**Fig. 7:**
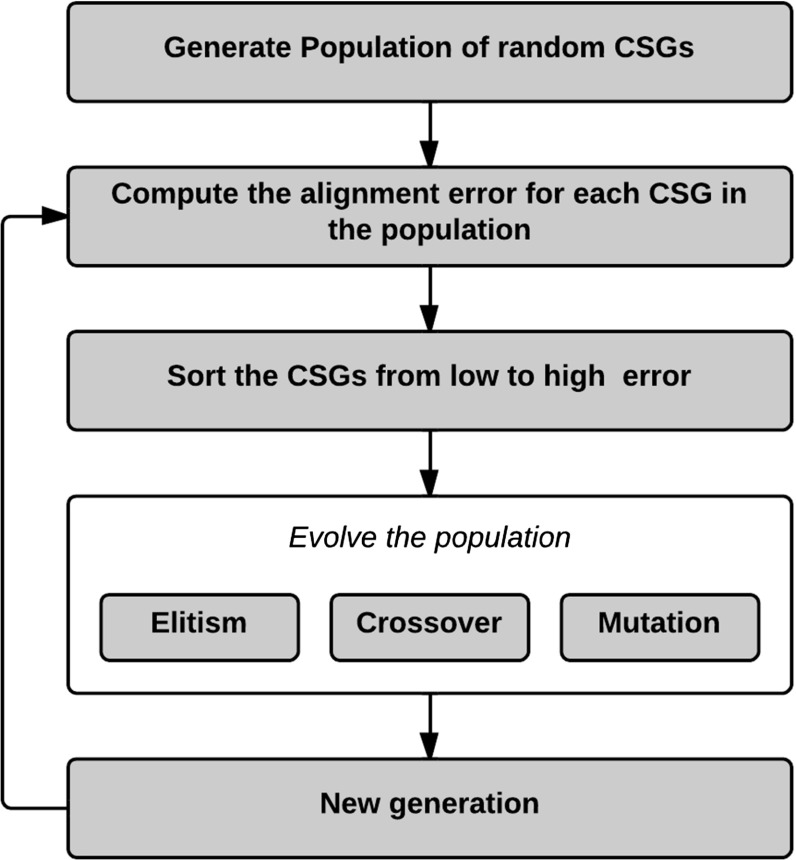
Overview of the genetic algorithm used in the CSG optimization algorithm

#### Genetic optimization

Given the sparse contact between the CSG and the bone, it is critical that the CSG configuration is tailored in such a way that it optimizes the fit and warrants stability and accuracy. For a given patient, we rely on an algorithm that uses a genetic optimization method driven by the previously defined objective function, which will be explained in this section. This solution allows us to handle the very large input parameter space (with around $2^{11\times 11}$ possible pin configurations), in which, given the current software and hardware resources, it would be impossible to evaluate all configurations iteratively. Although we refer to our pin-based CSG, most of this optimization strategy can, with minor modifications, be applied to other types of CSGs as well e.g., for hip replacement.

Genetic algorithms are inspired by natural evolution, in which fit individuals are more likely to survive [7]. Unfit individuals are removed by a selection process. The remaining population develops into new individuals via inheritance, crossover, and mutation. By iterating the selection and evolution steps, the individuals are likely to approach the local minima of the objective function. Figure 7 gives a schematic overview of the genetic optimization algorithm used in our approach.

**Fig. 8:**
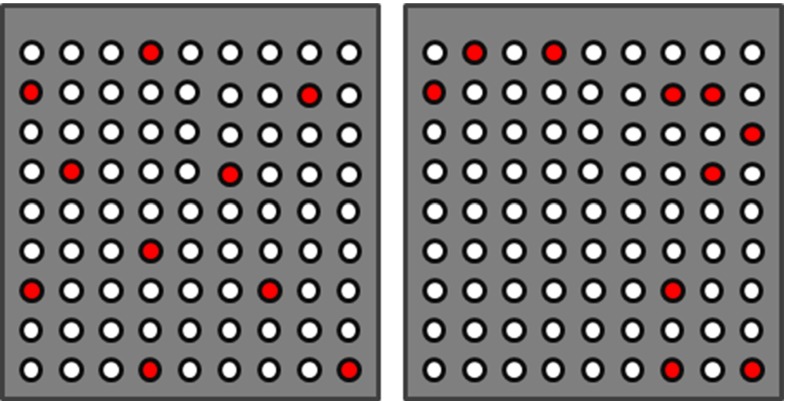
$\textit{Left}$ example of a CSG pin configuration using a Poisson distribution. $Right$ pin distribution as a result of random sampling, which leads to clumping of pins (exaggerated case). Although this pin distribution might work, there is a high probability that it will have a high alignment error, since there are no pins in the lower left corner

In our context, individuals correspond to different CSG configurations (see Fig. 2). In our case, each configuration csg consists of a set of active pins in the CSG (see Fig. 8). Initially, the CSG population consists of random active-pin distributions, which are established via a Poisson distribution to ensure a minimum distance between the pins and to avoid clumping, which leads to individuals with high alignment error that are unlikely to survive the genetic optimization. The pin insertion depth is determined automatically by moving the pins downward from the intended rest pose of the CSG until they collide with the surface of the bone.

Initially, our set consists of ten pins, which is a reasonable number to be configured manually. Introducing additional pins seems overly conservative, as in all test cases, ten led to solutions that respected the imposed accuracy constraints for practical use ($<$1.5 mm and $<$1$^\circ$). In fact, our algorithm always found solutions with even less pins while maintaining stability and accuracy.

**Fig. 9:**
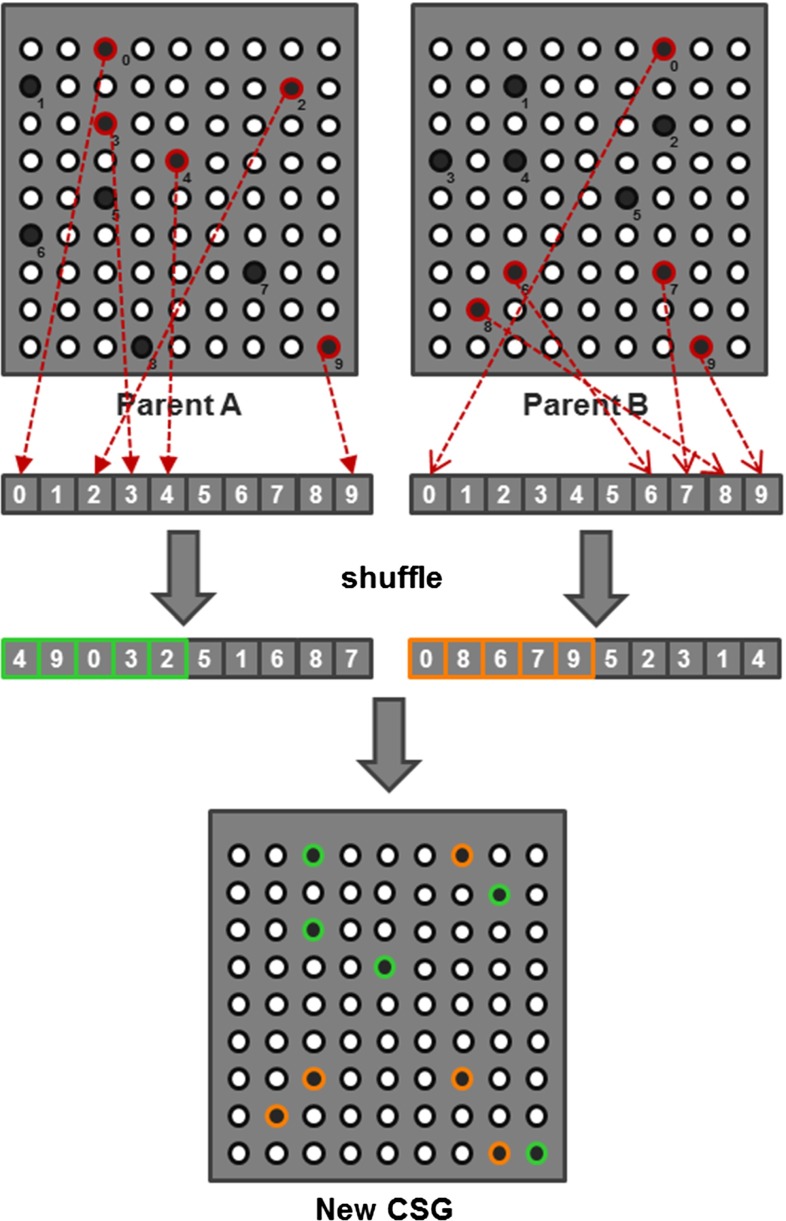
In the crossover stage, the configuration of two random CSGs (parent A and B) is combined to spawn a new CSG. A new CSG is formed by combining the pin IDs from two shuffled pin ID lists

**Fig. 10:**
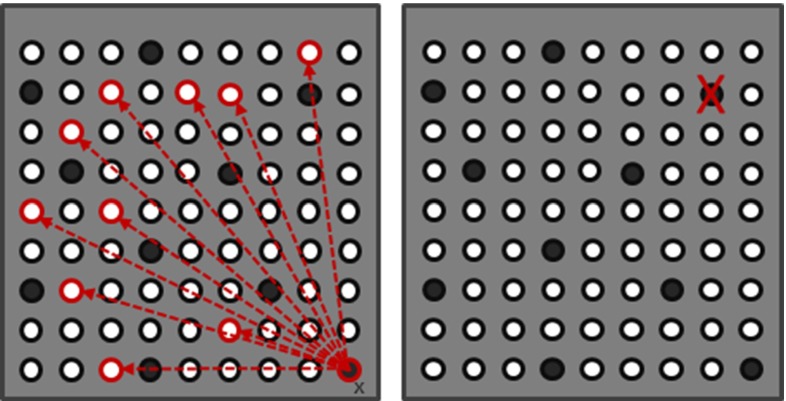
$\textit{Left}$ example of the single pin mutation strategy. Pin $x$ is deactivated, and an arbitrary other inactive pin is activated thereby creating a new CSG which is added to the population. $Right$ example of pin removal mutation. A new CSG is created by making a copy of the elite individual and removing a randomly selected active pin. The newly created CSG is added to the population

To evolve the set of current individuals, we apply elitism, crossover, and mutation. Elitism keeps the best individuals (elite) in the population to maintain their good properties. For crossovers, properties of two randomly chosen individuals are exchanged (see Fig. 9). Mutation means copying elite individuals and applying a slight configuration change. Precisely, a randomly chosen active pin is moved to a new location, (see Fig. 10). In order to reduce the probability of getting stuck in a local extremum, random CSGs are added to the population with a small probability.

Finally, we introduce a special mutation step with the goal of converging toward a minimal pin set; if an individuals’ error falls below a threshold $T$, as defined in the previous section, a second copy with one randomly removed pin is added to the population (see Fig. 10).

In theory, in an ideal case, only six pins might remain, which is the required number for a static equilibrium [13]. However, finding such a perfect configuration is particularly challenging and might not even be possible for all bone morphologies. It turns out that in practice, a minimum of eight well-distributed pins are required to reach a stable device placement (see Table 3).

The outline of our optimization strategy reads as follows:

1. Generate a CSG population of $n$ individuals;
2. For each individual, evaluate the objective function, see the section “CSG objective function”;
3. Sort the population based on alignment error in an ascending manner;
4. $c_\mathrm{e}$ percentage of individuals with the lowest error is propagated to the next generation without any modification (elitism);
5. For each individual whose error is below the given accuracy threshold, we add a copy with one removed pin to the population;
6. Complete the population to $n$ by performing crossovers and mutations and by inserting random individuals
  - $c_cn$ CSGs are created using crossover, where the parents are chosen proportionally to their error (individuals with low error are more likely to be chosen than individuals with high error);
  - The remaining fraction of $(1-c_c)n$ CSGs is used for mutation $(c_m n)$, and new individuals $c_nn$
7. If the best solution has been the same over $i$ iterations, we stop the algorithm; if it changed, we restart at step 2.

The following parameter set works well in practice: $n\,=\,50; c_e\,=\,6\,\%; c_c=0.5; c_n\,=\,0.1; c_m\,=\,0.4; i\,=\,50;T\,=\,0.5$ mm. Slight variations do not significantly impact the quality of the outcome.

#### Implementation and performance

**Fig. 11:**
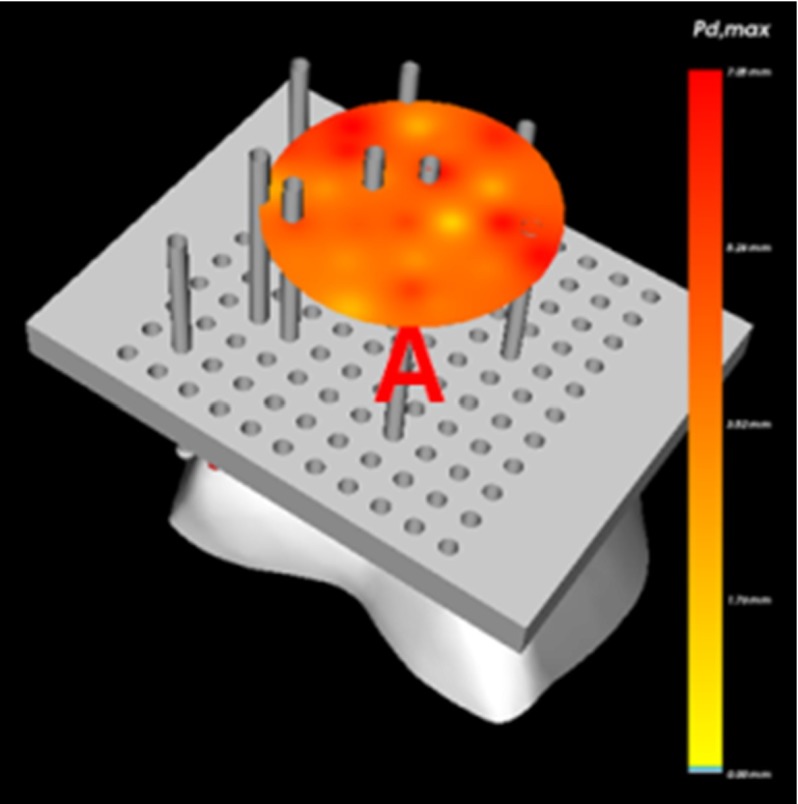
3D viewer for inspecting the guide animation

The optimization method described in this paper is implemented in C++, Python^1^ and OpenGL^2^, using the open source Bullet Physics^3^ Simulation API. Our optimization framework provides a complete interface for exploring all aspects of the optimization process, meaning that the end user can see how CSGs evolve via the genetic optimization. For all CSGs, the user can inspect the configuration and interaction with the bone from different directions. The maximum pin drift associated with the directions is temporally visualized via a disk (see Fig. 11). Furthermore, the system allows the user to make small changes to the optimized CSG, in order to investigate the impact of a change on stability.

Table 2 shows the timings of the optimization routines for various CSGs applied to the distal femur model. Although the timings in Table 2 are considerable, there is still room for improvement, as our primary focus was the development of the optimization technique itself, and not particularly its performance. Since the physical simulation is entirely independent, it is well suited for a multi-threaded environment, resulting in a roughly linear speedup in the number of cores of the system. Further, using graphics hardware for the physical simulation (e.g., for collision detection) might result in a significant speedup, as evidenced by recent graphics engines, such as Optix [18].

**Table 2:** List of computer-optimized CSGs and the time it took to run the genetic optimization

Exposure	No. of generations	Time
Full	49	1:24:43
Full	26	0:39:47
Full	26	0:22:25
Limited	26	0:37:04
Limited	45	0:48:06
Limited	16	0:20:30
Limited${^\mathrm{a}}$	189	1:29:25
Limited${^\mathrm{a}}$	200	2:56:18
Limited${^\mathrm{a}}$	192	5:01:23

$^\mathrm{a}$ The number of pins is minimized

### Experiments

The goal of our experiments is threefold. First we want to determine the accuracy and reliability of our optimization method compared to the manual method, taking into account full and limited surgical exposure. Second, we want to see whether our optimization method works with varying bone geometries. Third, we want to verify whether our pin minimization method leads to accurate and consistent results. In the next sections, we describe our experimental setup, which CSG-bone combinations were tested, and how we performed the measurements.

#### Setup

**Fig. 12:**
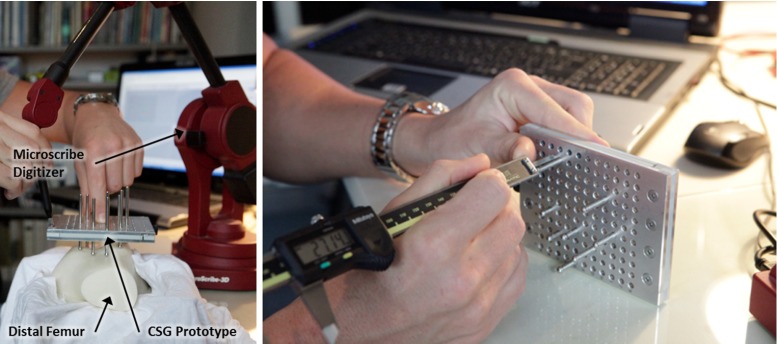
Photographs of the experiment setup. $\textit{Left}$ the 3D print of the distal femur has been draped with a cloth to mimic a real operating scenario. After the CSG has been placed on the 3D print of the distal femur, four points on the CSG prototype are digitized using the 3D point digitizer in order to derive a transformation matrix and subsequently translational and rotational errors. $Right$ CSG prototype is manually configured using a digital caliper

The experimental setup as shown in Fig. 12 comprises a prototype of the pin-based CSG, a 3D printed distal femur and a 3D point digitizer (Microscribe^4^). The bone model is scaled 1.5 times to minimize any potential errors due to the limited resolution of the fused deposition modeling printing technology ($0.17$ mm) and errors in 3D point digitization. To evaluate the configuration of a CSG, we measured its precise location and orientation after placing it on the bone, see the “Measurement method” section.

**Fig. 13:**
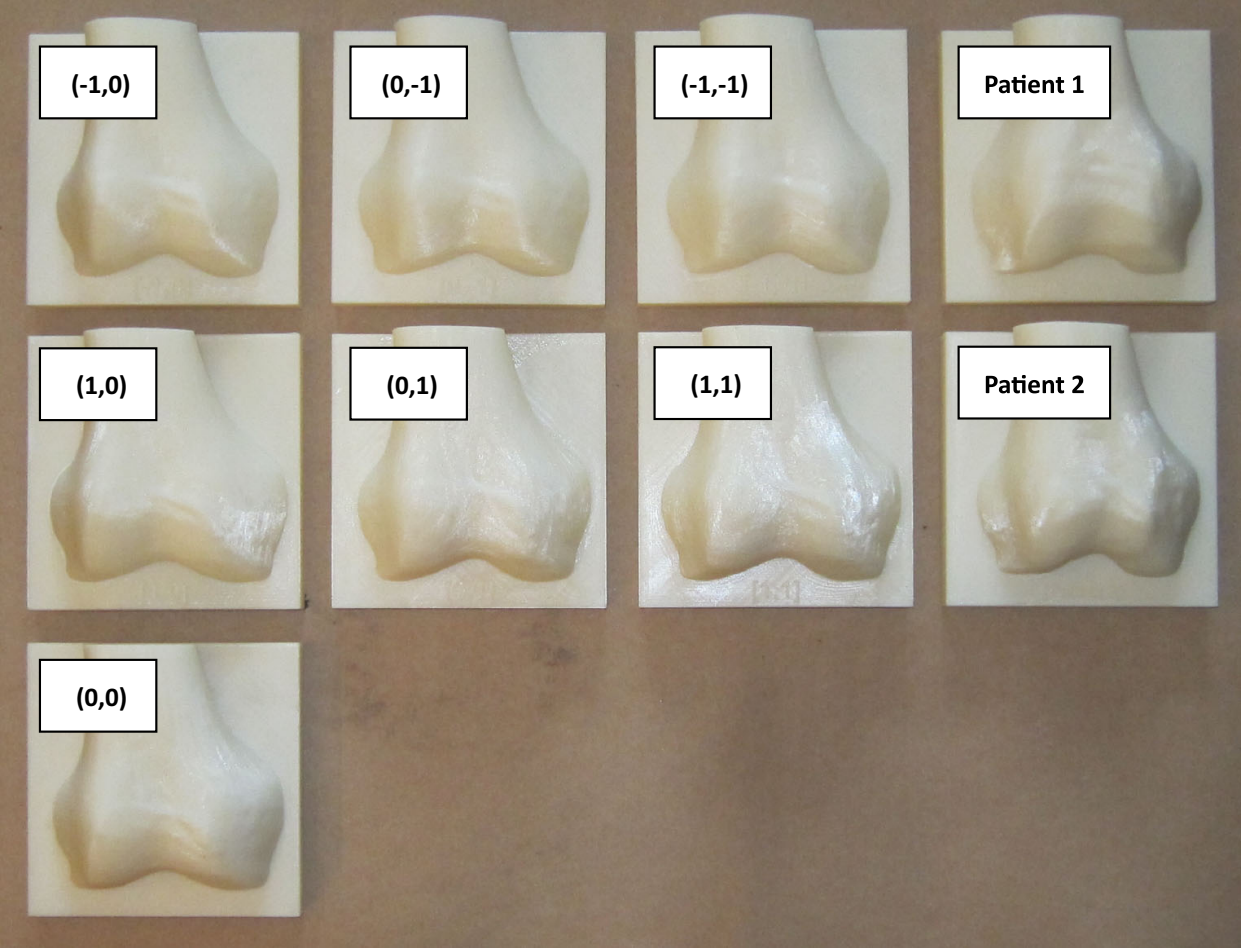
3D printed distal femora that were used in the experiments

#### CSG configurations

We tested the prototype of the pin-based CSG on nine 3D printed distal femora (see Fig. 13). Two are based on actual patient data, and the remaining seven are generated by an Active Shape Model (ASM) which was built from a training set of 62 distal femora as described in [2]. Shapes were extracted from the ASM by varying the first two modii of variation. We created a mean femur and six extremes of the first two modii of variation (see Table 3).

**Table 3:** Overview of the CSG-bone combinations that were tested

Bone			Exposure	Type	No. of CSGs
ASM	0	0	Full	Manual	9
	0	0	Full	Optimized	3
	0	0	Limited	Manual	9
	0	0	Limited	Optimized	3
ASM	$-$1	0	Limited	Optimized	2
ASM	1	0	Limited	Optimized	2
ASM	0	$-$1	Limited	Optimized	2
ASM	0	1	Limited	Optimized	2
ASM	$-$1	$-$1	Limited	Optimized	2
ASM	1	1	Limited	Optimized	2
ASM	0	0	Limited	Optimized${^\mathrm{a}}$	3
Patient 1			Limited	Optimized	2
Patient 2			Limited	Optimized	2

CSGs were tested on bones from actual patient data and bones extracted from an ASM, the input modii of variation are mentioned in the second, and third column

$^\mathrm{a}$ Pin count was also optimized (eight, eight, and nine pins, respectively)

**Fig. 14:**
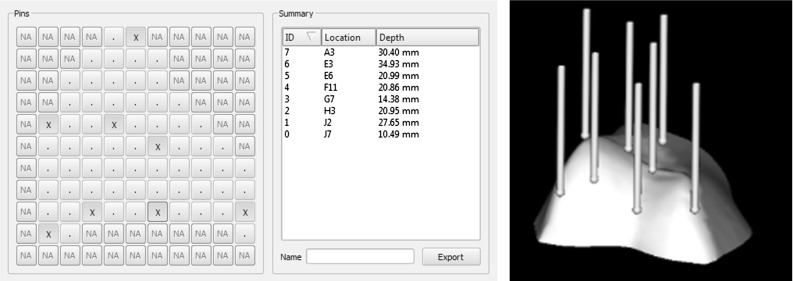
Interface for manually creating a pin-based CSG configuration. $\textit{Left}$ user interface for choosing a pin configuration, in this case, the user can only pick a limited amount of pins because the exposure is limited. $Right$ Visualization of the pins on the surface of the cartilage/bone in the planned alignment

The manually configured CSGs from Table 3 were generated by nine participants (age 24–62). Participants were divided into three groups: (a) three untrained participants without special a priori knowledge of human anatomy, (b) four medical visualization students with prior anatomical knowledge, but no surgical experience (although one even has a background as a radiology assistant), and (c) two expert orthopedic surgeons (approximately 15 and 30 years of surgical experience). The concept of the pin-based CSG was explained to the participants, stressing the importance of alignment reproducibility and stability of the CSG when docked onto the bone. They were asked to create two pin configurations (based on full and limited exposure) that would optimize the placement of the CSG in its equilibrium state (the smallest translational and rotational error with respect to the planned alignment). To facilitate this task, participants were given the option to use our computer program to set active pins using a mouse and to see the corresponding CSG device in the intended equilibrium state with all active pins in contact with the bone (see Fig. 14). The experiments were performed under no time pressure; each participant could use as much time as wanted and had as many attempts as needed to setup a configuration. Up to ten pins were allowed to be placed on the device, despite the possibility to use less, all participants used all pins. The experiments started with a quick demonstration of an ad hoc configuration and a short explanation of the simple computer program to set and investigate the pin combination. Participants took between one and three minutes to create a pin configuration.

#### Measurement method

For each CSG in Table 3, we used a digital caliper ($\pm 0.01$ mm) to adjust the pin depth to carefully reproduce each CSG configuration (see Fig. 12). Next, it was deployed ten times on the 3D printed distal femur. In order to measure how much the CSG deviates from the planned position and orientation, two point-paired registrations are performed by digitizing reference points (known in the virtual and the real world) on the CSG and the 3D printed distal femur using the 3D point digitizer (see Fig. 12).

While in theory, three reference points are sufficient for point-paired registration, for practical reasons and to increase accuracy, we obtained four reference points on the CSG (located at the corners of the device). Given the resulting registrations, the homogeneous matrices describing the position and orientation can be computed. From this transformation matrix, we derive the distance between the intended and the actual location, and the angle between the intended and actual orientation vectors to verify the accuracy of the alignment.

## Results

**Fig. 15:**
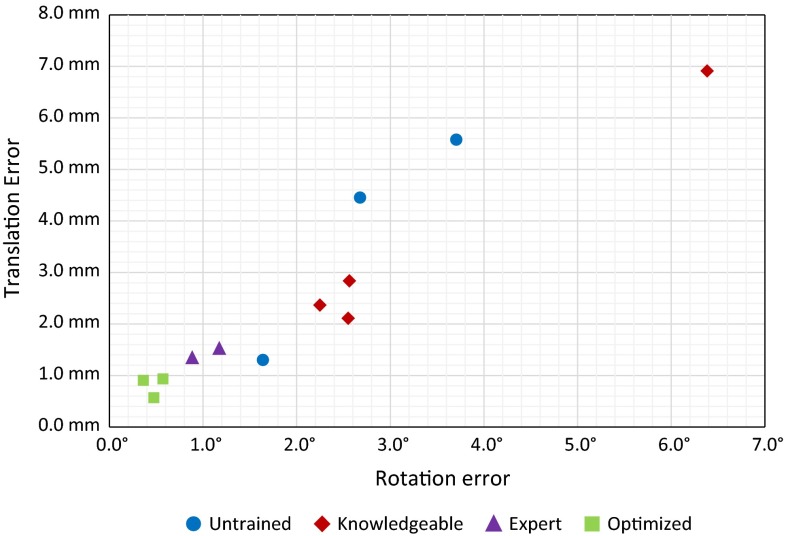
Alignment errors as a result of placing manually configured as well as computer-optimized CSGs (full exposure) on the mean distal femur from our ASM

**Fig. 16:**
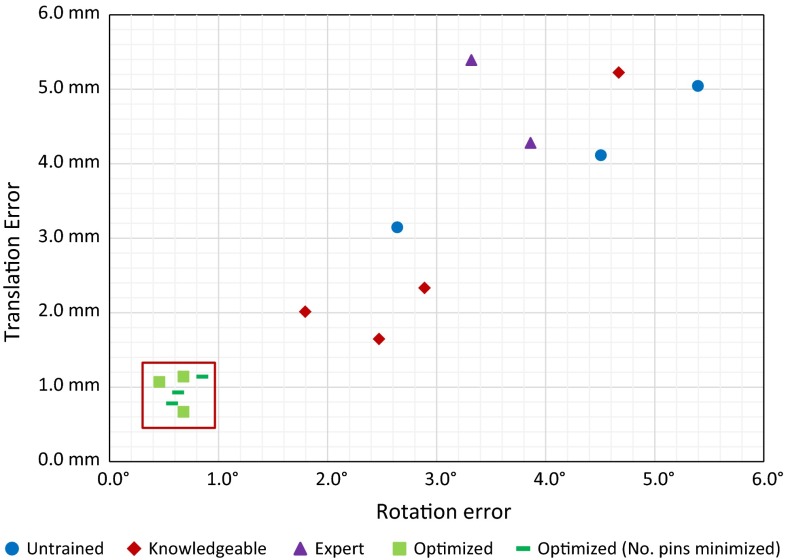
Alignment errors as a result of placing manually configured as well as computer-optimized CSGs (limited exposure) on the mean distal femur from our ASM. Three additional CSGs have been tested with a minimized number of pins

**Fig. 17:**
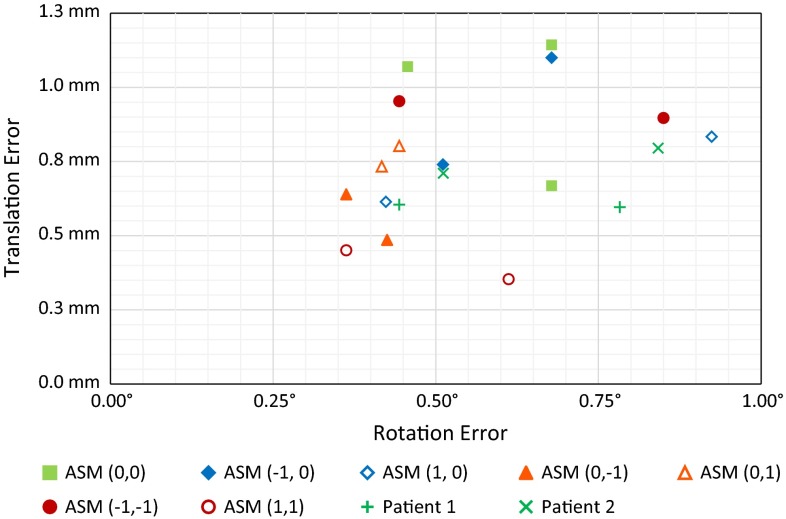
Alignment errors as a result of placing computer-optimized CSGs (limited exposure) on nine  distal  femora, two based on real patient data and seven based on shapes derived from our ASM

The results from the experiments are depicted in Figs. 15, 16 and  17. In these scatter plots, each marker represents a single CSG configuration that is tested on a 3D printed distal femur, and the position of the marker denotes the maximum translation and rotation error that were measured during the experiments.

Figures 15 and 16 indicate that the optimized CSG configurations always outperform manually configured CSGs. The optimization process will always ensure that the deviation threshold is respected—here 1.5 mm and $<$1$^\circ$. Further, the computer-optimized CSGs were successfully placed in each trial, indicating that there is sufficient haptic feedback and stability to warrant a proper alignment. In contrast, there is a significant spread in alignment error among manually configured CSGs.

The surgeon-defined CSGs are superior to those of the novices for the full exposure, which might be due to the experts’ substantial knowledge about human anatomy and morphology of the femur, and these full exposure CSGs could actually be considered acceptable. Nonetheless, there is no guarantee that such manual definitions will perform well, especially considering the spread of the various samples.

The situation actually changes drastically, when investigating the limited exposure scenario, which can also be considered more realistic. Here, the configuration process is more complex because certain surface regions need to be avoided. All manually defined devices perform significantly worse, including the surgeons, and the difference to our optimized CSGs becomes very obvious—see the cluster of optimized guides (all within 1.2 mm translational error and $0.9^\circ$ rotational error) versus the surgeon-defined CSGs, which now belong to the worst performing CSGs.

The results from the experiments clearly indicate that manual CSG configuration is a delicate and complex task with often poor results, while the optimization framework consistently leads to reproducible and reliable configurations, even in cases where the number of pins is minimized (see Fig. 16).

In an additional verification step, we asked three participants to redo the user study three times, but none managed to improve their manual results significantly ($<$1.2 mm and $<$0.9$^\circ$ compared to the optimized). This fact further underlines that configuring CSGs is not intuitive, even after experimenting for a considerable amount of time.

Finally, Fig. 17 shows the errors of 19 computer-optimized CSGs on a variety of bone shapes. The deviation threshold is respected by all samples, which strongly suggests that our optimization method is robust to varying input geometry as well.

## Discussion and conclusions

Alignment of prosthetic implants in joint replacement surgery has a significant impact on the survival of orthopedic implants [3, 11]. Besides other factors, especially mal-alignment can lead to aseptic loosening, premature failure, and impaired range of motion [31]. Computer navigation (CT-based and CT-free) and patient-specific templating have improved the accuracy of alignment and reduced the chance of outliers [12, 16].

Our study focusses on CSGs, and in particular on the computer-assisted definition of guide input parameters to warrant a reliable alignment during surgery. No current studies exist that describe such a method for CSG optimization. Predicting the actual alignment of CSGs is difficult and requires knowledge about the physical interactions between the CSG and the host bone. Non-surgeons, as well as experienced orthopedic surgeons, struggle with the task. Not only is the alignment accuracy of human-defined CSGs low, but there is also a significant spread between subjects. In our method, we currently do not address the effect of imaging modality choices (CT/MRI) [34], their effect on bone reconstruction and subsequently alignment accuracy. The topic of uncertainty with respect to bone reconstruction and manual guide configuration accuracy is considered a topic on its own and could lead to a follow-up study, involving also a clinical pilot study.

In summary, we introduced a novel computer-assisted method to configure CSGs and predict their reliability during surgery. It will help surgeons to follow the planned alignment more closely, and ultimately lead to an improved surgical outcome. For the patient, this means less postoperative pain, improved function, and longevity of the joint. Although our method is validated on the knee joint, it is certainly not restricted to this application, since our optimization procedure is more general and can handle any arbitrary types of bone geometry. Furthermore, our simulation framework supports different guides as input, which makes it also interesting for PST designers. The pin-based CSG we used in this article can be applied to other joints as well, taking into account the specific joint anatomy and accessibility. The design of new CSGs and exploring variations of the current one are interesting areas of future work. These aspects illustrate several of the advantages and the generality of our approach, which makes it widely applicable.

## Footnotes

1
https://www.python.org/.

2
http://www.opengl.org/.

3
http://bulletphysics.org.

4
http://www.3d-microscribe.com/.
